# Neuropsychiatric symptoms of cholinergic deficiency occur with degradation of the projections from the nucleus basalis of Meynert

**DOI:** 10.1007/s11682-016-9631-5

**Published:** 2016-10-27

**Authors:** Jan Willem van Dalen, Matthan W.A. Caan, Willem A. van Gool, Edo Richard

**Affiliations:** 1 Department of Neurology, Academic Medical Center, University of Amsterdam, Meibergdreef 9, 1105 AZ Amsterdam, Netherlands; 2 Department of Radiology, Academic Medical Center, University of Amsterdam, Amsterdam, Netherlands; 3 Department of Neurology, Radboud University Medical Center, Nijmegen, Netherlands

**Keywords:** Dementia, Neurodegeneration, Tractography, Acetyl cholinesterase, White matter, Behavioral symptoms, White matter hyperintensities

## Abstract

This study aims to evaluate the relation between a cluster of neuropsychiatric symptoms related to cholinergic deficiency and degradation of the cortical cholinergic projections which project from the nucleus basalis of Meynert to the cerebral cortex. An atlas of the pathway from the nucleus basalis to the cortex (NbM cortical pathway) was constructed using diffusion tensor imaging and tractography in 87 memory clinic patients. Structural degradation was considered to be represented by lower fractional anisotropy (FA) and higher mean diffusivity (MD). Neuropsychiatric symptoms were assessed using the Neuropsychiatric Inventory. A predefined cluster including agitation, anxiety, apathy, delusions, hallucinations, and irritability was labeled as the cholinergic deficiency syndrome (CDS). In regression analyses, lower FA and higher MD in the NbM cortical pathway were associated with CDS symptoms but not with other neuropsychiatric symptoms. These associations were independent of cerebral atrophy and overall FA or MD. There was no association between interruption of the NbM cortical pathway by white matter hyperintensities and CDS symptoms. Cox regression suggested a trend for higher mortality with lower FA in the NbM cortical pathway may exist. These findings provide anatomical support for the hypothesis that degradation of the cholinergic projections from the nucleus basalis of Meynert may lead to a distinct clinical syndrome. Future studies could use our results to test the utility of assessing NbM projection integrity to identify patients who may benefit from cholinergic treatment or with a worse prognosis.

**Electronic supplementary material:**

The online version of this article (doi:10.1007/s11682-016-9631-5) contains supplementary material, which is available to authorized users.

## Introduction

The cholinergic innervation of the neocortex stems almost exclusively from the nucleus basalis of Meynert (NbM), located in the basal forebrain (Saper et al. 2010; Mesulam 2013; Gratwicke et al. 2013). Atrophy of the basal forebrain cholinergic system is a progressive feature in normal aging, aggravated in mild cognitive impairment and pervasive from the onset in Alzheimer’s and other dementias (Grothe et al. 2012; Teipel et al. 2011; Schliebs and Arendt 2011). Loss of cortical cholinergic innervation is thought to play an important role in the development of psychiatric and behavioral symptoms (Pinto et al. 2011). Experimental and clinical observations have led to the hypothesis that a specific subset of neuropsychiatric symptoms is susceptible to cholinergic medication (Lemstra et al. 2003; Pinto et al. 2011). This cluster of symptoms, termed “Cholinergic Deficiency Syndrome” (CDS), was hypothesized to be evoked by impaired cortical cholinergic stimulation, causing loss of attention, concentration, and capacity to discern stimuli, provoking clinical symptoms including agitation, anxiety, apathy, delusions, hallucinations and irritability (Lemstra et al. 2003; Pinto et al. 2011).

From the NbM in the basal forebrain, cholinergic fibers travel towards the cortex together with other, non-cholinergic, projections from the basal forebrain and mesencephalon (Saper et al. 2010). These projections form a medial and lateral bundle from which axons emanate and fan towards the cortex (Mesulam 2013; Selden et al. 1998). According to the CDS hypothesis, disruption of the cholinergic NbM projections may incite CDS symptoms, by chronically interfering with cortical cholinergic innervation. The diversity of the symptoms associated with the CDS, may be explained by the different roles the reciprocal NbM projections fulfill throughout the cerebrum by serving more fundamental roles of detection, selection, discriminating and processing of stimuli (Lemstra et al. 2003; Pinto et al. 2011; Klinkenberg et al. 2011). Cholinergic stimulation of the hippocampal area seems important for short-term information acquisition, of the amygdala for anxiety regulation, and of the somatosensory areas for stimulus discrimination (Klinkenberg et al. 2011). Disruption of the NbM projections to the limbic system may lead to emotional changes, to the cingulate and prefrontal cortex to motivational and executive symptoms, and to the occipital cortex to hallucinations (Pinto et al. 2011; Klinkenberg et al. 2011; Liu et al. 2015). However, most likely behavioral symptoms rely on synchronized activity regulation of a wide range of neuronal networks and cannot be attributed to dysregulation of single, clearly delimited brain regions (Klinkenberg et al. 2011).

One cause of disruptions of the NbM projections may be white matter hyperintensities of presumed vascular origin (WMH), a common finding on brain magnetic resonance imaging (MRI) in older persons (Gouw et al. 2011; Moran et al. 2012; Debette and Markus 2010; Schmidt et al. 2011). Pathologically, WMH are heterogeneous anomalies thought to represent various degrees of ischemic white matter damage (Gouw et al. 2011; Moran et al. 2012; Schmidt et al. 2011). Their clinical correlates include cognitive and neuropsychiatric symptoms (Gouw et al. 2011; Moran et al. 2012; Debette and Markus 2010; Schmidt et al. 2011; Berlow et al. 2010), and may be spatially dependent (Tighe et al. 2012; Mortamais et al. 2013; Bocti et al. 2005; Bohnen et al. 2009). For example, WMH interfering with the cholinergic NbM projections may incite symptoms of cholinergic deficiency (Bocti et al. 2005; Bohnen et al. 2009). Although WMH severity in regions likely to contain NbM projections has been associated with cognitive impairment, accurate identification of these projections is difficult (Bocti et al. 2005; Bohnen et al. 2009; Kim et al. 2012; Behl et al. 2007). This makes further delineation of cholinergic brain-behavior relations problematic.

Previous studies have identified the cholinergic projections using visual rating scales (Bocti et al. 2005; Behl et al. 2007). Other studies have used diffusion tensor MRI to identify the NbM and linked NbM atrophy in MCI patients to memory and attentional deficits, cognitive decline and conversion to Alzheimer’s disease (Grothe et al. 2016; Brueggen et al. 2015; Grothe et al. 2013; Teipel et al. 2011; Grothe et al. 2010). The correlates of loss of integrity to the NbM projections have been little studied, although one study was able to reconstruct the medial branch of the pathway passing through the cingulum using diffusion tensor MRI (Hong and Jang 2010). This study aims to assess the clinical manifestations associated with structural degradation of the cortical NbM projections in memory clinic patients evaluated for cognitive impairment. The pathways containing these projections (NbM cortical pathways) are reconstructed in vivo using probabilistic tractography in diffusion weighted MRI data (Jones 2008). White matter degradation is considered to be captured by lowered fractional anisotropy (FA) and increased mean diffusivity (MD) within the reconstructed projections (Jones et al. 2013). We hypothesized that such changes in FA and MD in the NbM projections are associated with the clinical symptoms of the CDS and that WMH mediate this association through interference with cholinergic projections. Also, since NbM degeneration has been associated with more advanced disease (Grothe et al. 2013), it may be a marker of a worse prognosis. In an exploratory setting, we evaluated this possibility by assessing whether NbM degeneration was associated with increased mortality.

## Material and methods

### Population and clinical assessment

Participants were derived from a prospective cohort of patients referred to the memory clinic of the Amsterdam academic medical Centre for the evaluation of suspected cognitive impairment. In addition to regular outpatient assessment by a neurologist or geriatrician, they underwent a standardized protocol including several questionnaires, neuropsychological testing and MRI as part of the Dutch Parelsnoer neurodegenerative disease study (Talmon et al. 2008; Aalten et al. 2014). Inclusion criteria for this study were the presence of cognitive complaints, a clinical dementia rating of 0.5 or 1, a mini mental state examination (MMSE) score > 19 at the time of first visit to the memory clinic, and a complete data set including MRI and neuropsychiatric assessment.

In addition to a general physical and neurological examination, an extensive medical and social history was obtained from patients and informants by an experienced neurologist or geriatrician. A trained research nurse administered several questionnaires assessing clinical characteristics. Global cognition was rated using the MMSE and the clinical dementia rating scale. Neuropsychiatric symptoms were assessed using the 12-item neuropsychiatric inventory (NPI). A neuropsychological test battery was administered including for mermory: the 15-word auditory verbal learning test (Brand and Jolles 1985; Rey 1958) and the digit-span of the WAIS III (forward and backward) (Wechsler 1997), for associative visual learning the short version of the visual association test (Lindeboom et al. 2002), for verbal fluency: the 60 s animal fluency test (Lezak 1995), and for information processing speed: the 60 s letter digit substitution test (van der Elst et al. 2006), the Stroop color word test (10 × 10 items, 4 colors) (Stroop 1935; van der Elst et al. 2008) and the trail making test (TMT) part a and B (Reitan 1958). T-scores for these tests were calculated according to Dutch normative data adjusted for sex, age and education. Taking this information into account, a clinical diagnosis was made by a multidisciplinary council consisting of experienced neuropsychiatrists, neurologists and geriatricians based on DSM-IV criteria (APA 1994). Dementia subtypes were based on standardized clinical criteria for Alzheimer’s disease (NINCDS-ADRDA (APA 1994, McKhann et al. 1984), vascular dementia (NINDS-AIREN criteria) (Roman et al. 1993), frontotemporal dementia (Neary et al. 1998), and Lewy body dementia (McKeith et al. 1996). Survival or date of death was registered at telephone follow-up three years after baseline or through the Dutch municipal personal records database. The study was approved by the Amsterdam academic medical Centre institutional review board and all participants provided written informed consent.

### Definition of the CDS

Our main outcomes were: 1) number of symptoms of the cholinergic deficiency syndrome (CDS), and 2) presence of the CDS, operationalized as the presence of more than one CDS symptom. CDS symptoms were defined as the following items of the NPI: agitation, anxiety, apathy, delusions, hallucinations and irritability (Pinto et al. 2011). Symptoms were qualified as present if the product of the NPI symptom frequency and severity score was >3 (Lyketsos et al. 2002). To contrast CDS symptoms with any other neuropsychiatric symptoms, the number of NPI symptoms *not* included into the CDS and the presence of >1 *non*-CDS symptom were identically analyzed as outcomes.

### MRI acquisition

Imaging was performed on a 3.0 Tesla MRI system (Philips Intera, Best, the Netherlands) with an 8-channel head coil. Diffusion weighted MRI was performed by multi-slice spin echo single shot echo-planar imaging, with TE/TR = 94/6105 ms; four averaged volumes with diffusion sensitivities of *b* = 0 s/mm^2^; 32 diffusion gradient directions with *b* = 1000 s/mm^2^; 48 continuous (no inter-slice gap) slices, slice thickness 3 mm, field of view = 229 × 229 mm^2^; acquisition matrix = 128 × 128; acquisition voxel size = 1.79 × 1.79 × 3 mm. Furthermore, a gradient echo 3D FFE, T1-weighted, sagittal sequence was used with TE/TR = 3.5/9 ms, field of view = 256 × 232 mm^2^, scanning matrix = 256 × 231, flip angle = 8°, voxel size = 1x1x1 mm. Also, a 2D FLAIR sequence was run, with TE/TR = 100/11,000 ms, TI = 2600 ms, field of view = 230 × 183 mm, 48 slices of 3 mm thickness, NSA = 2.

Data were anonymized prior to analysis. Diffusion tensor imaging data was preprocessed using in-house developed software written in Matlab (The MathWorks, Natick, MA), executed on the Dutch e-Science Grid (www.biggrid.nl), using a web interface to the e-Bioinfra gateway (Shahand et al. 2013). Head motion and deformations induced by eddy currents were corrected for by an affine registration of the diffusion weighted images to the non-diffusion weighted image. Gradient directions were corrected by the rotation component of the transformation. Diffusion weighted images were resampled isotropically and Rician noise was reduced using adaptive noise filtering (Caan et al. 2010). Diffusion tensors were estimated in a non-linear least squares sense. From the tensors, FA and MD maps were computed.

### Tractography

**Fig. 1:**
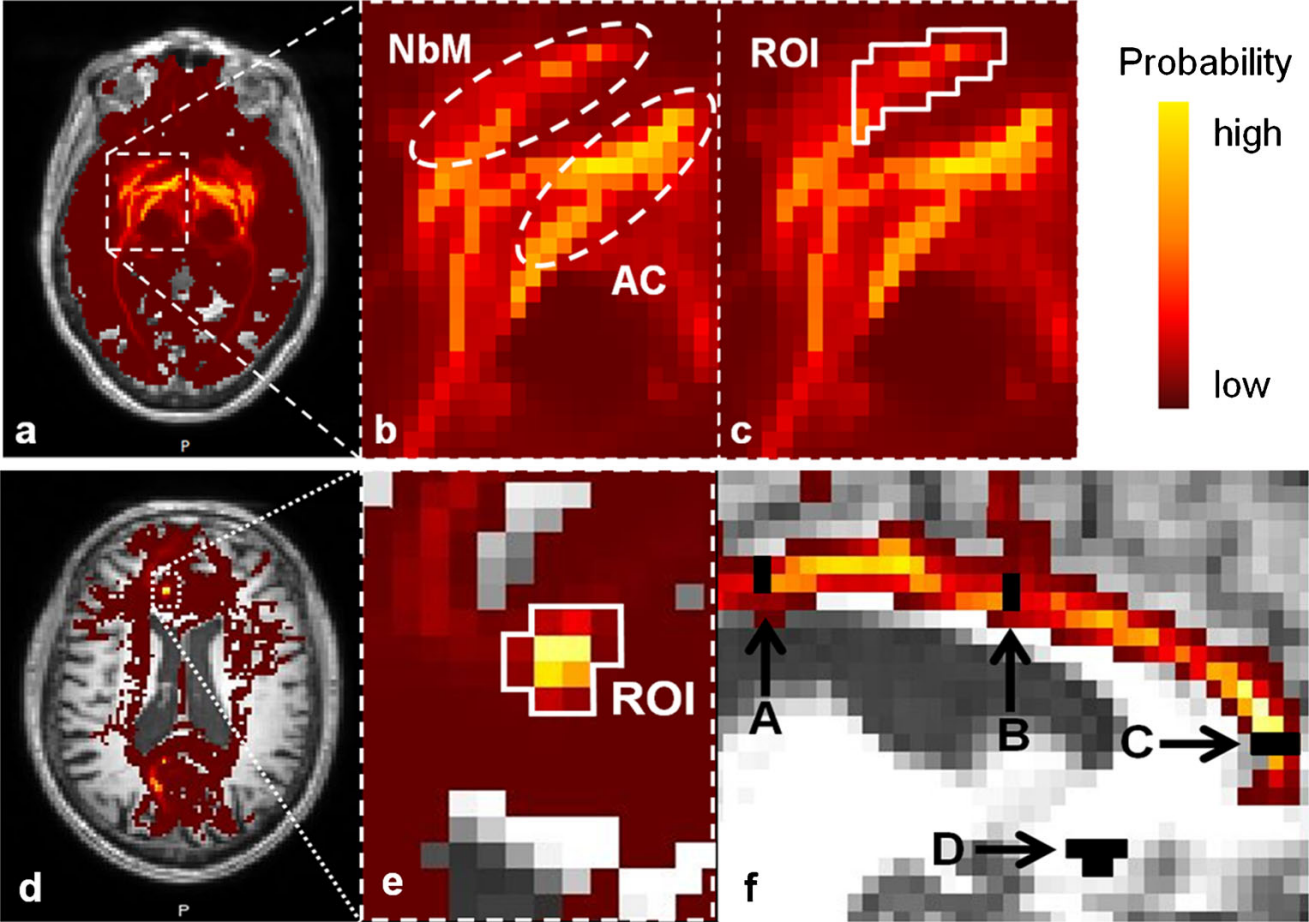
ROI placement for the lateral (*top row*) and medial (*bottom row*) tracking technique. Connectivity map of the region encompassing the NBM projected onto the T1 scan of the same patient. Voxels with a high probability of containing fiber tracts are depicted in light yellow, resolving into dark red as probability decreases. *Top row*: **a** Axial slice, slightly caudal to the anterior commissure. **b** Detailed view of the region of the NbM. NbM: Track identified as originating from the basal nucleus (NbM). AC: Track identified as originating from the anterior commissure. **c** Detailed view of the region of the NbM. ROI: seed ROI for definitive fiber tracking, covering the fibers suspected to originate from the NbM. *Bottom row*: **d** Axial slice at the middle of the genu of the corpus callosum. **e** Detailed view of the cingulate gyrus at the genu of the corpus callosum. The definitive seed ROI was placed on the tracts identified by fiber tracking of the cingulate gyrus **f** Sagittal slice through the left cingulate gyrus, A: posterior cingulate gyrus waypoint, B: anterior cingulate gyrus waypoint, C: medial nucleus basalis of Meynert (NbM) seed, D: lateral NbM seed (top row c), used as third waypoint for the medial tracking technique

Tractography estimates the course of neuronal fiber tracts crossing a pre-defined “seed” region. We performed probabilistic tractography with additional processing (BEDPOSTX) for crossing fibers using Functional Software Library (FSL) software (Jenkinson et al. 2012). From each seed voxel 5000 streamline samples were generated (step length: 0.5 mm, curvature threshold: 0.2). This produced a connectivity map for each seed region, with voxel intensity representing the relative frequency streamline samples included that voxel (Fig. 1). Higher intensity voxels are deemed more likely to contain neural tracts connected to the seed region.

The cholinergic NbM projections run along two distinct pathways: 1) the medial pathway predominantly through the cingulate gyrus, and 2) the lateral pathway towards the temporal lobe and towards posterior along the extreme capsule (Mesulam 2013). To optimize NbM localization in each individual, we adapted earlier protocols identifying mainly the lateral (Teipel et al. 2011), and medial projections (Hong and Jang 2010). Two seed regions of interest (ROIs) were defined, in native diffusion space, on the FA and color coded orientation maps. The first was placed on the lateral side of the NbM (lateral ROI), the second at the genu of the anterior cingulate gyrus (medial ROI).

To accurately place the lateral ROI on tracts originating from the NbM, first, exploratory tractography of the region encapsulating the NbM was performed. Probablistic fiber tracking was performed from a box-shaped seed ROI based on previous findings (Teipel et al. 2011), extending from the posterior of the anterior commissure at the midline, 9 mm anteriorly, 15 mm posteriorly, 15 mm ventrally, and laterally to the sagittal plane at the lateral border of the amygdala. On the resulting connectivity map, the lateral NbM tracts were identified as areas of high connectivity located anteroventrally to the tracts crossing the anterior commissure (Fig. 1b). The definitive lateral seed ROI was placed on these tracts (Fig. 1c). This ROI extended from medioventral, superior to the plane of the anterior commissure and laterally to the sagittal plane at the lateral border of the amygdala, and only incorporated tracts appearing to originate from the NbM. From this ROI, definitive lateral fiber tracking was performed. To restrict the reconstructions to the cortical NbM projections and minimize false positives, tracking results crossing the midline, brain stem, hypothalamus, or optic chiasm, were excluded with exclusion masks defined using the T1-weighted scan and primary connectivity map.

For the medial ROI, we first performed exploratory tractography of the tracts through the cingulate gyrus using two seed ROIs placed on the cingulate gyrus in the coronal plane, at the level of the anterior and posterior commissure. Using the obtained connectivity map, a new seed ROI was placed in the axial plane, on the fibers through the cingulate gyrus, at the midline of the genu of the corpus callosum (Fig. 1e, f: C) (Hong and Jang 2010). From this ROI, definitive medial fiber tracking was performed. To minimize false positives, three waypoint ROIs were used: two were placed on the cingulate gyrus tract in the coronal plane above the anterior and posterior commissure (Fig. 1f: A and B), the third was the definitive lateral seed ROI (Fig. 1f: D). Only tracking results intersecting at least one waypoint were retained. Exclusion masks were not employed.

ROIs were identified manually by one rater according to strict specifications to maximize reproducibility. Intra-rater reproducibility was assessed in 10 random scans. Tracking was performed separately for the medial and lateral ROIs and separately in each hemisphere. Maps of the NbM cortical pathway reconstructed from the lateral and medial ROI were combined to form one NbM projection map for each patient.

### Generation of NbM cortical pathway atlas

To avoid statistical biases introduced by individual variance in reconstruction accuracy (Jbabdi and Johansen-Berg 2011), individual results were combined to form a population atlas of the NbM cortical pathway, on which analyses were based. FA volumes were non-linearly registered to standard space (Andersson et al. 2007a, 2007b). Normalized individual tracking results and MD maps were warped accordingly. The population atlas was created by averaging the warped tracking results. To discard spurious results, connectivity map minimum intensity thresholds were arbitrarily set at 5 % of the maximum intensity (Groppa et al. 2014; Galantucci et al. 2011), at which they showed good resemblance to anatomical descriptions of the NbM projections (Selden et al. 1998). Since the NbM projections fan out towards the cortex, white matter degradation more proximal to the NbM may have a more general disruptive effect on cortical stimulation than more distal degradation. To evaluate this possibility, we divided the reconstructed NbM pathway into proximal, intermediate, and distal regions. For this purpose, a ROI drawn around the NbM was expanded over the pathway in steps of 1 voxel, until its overlap was complete. The proximal, intermediate and distal segments were based upon tertiles of the number of steps needed to cover the entire pathway.

### Brain structure parameters

For each patient, mean FA and MD in the whole, the proximal, intermediate, and distal segments of the NbM pathway were calculated. Whole brain mean FA and MD (overall FA and overall MD) were computed to allow comparison with global FA and MD effects. Using Slicer 3D (Fedorov et al. 2012), WMH were segmented on FLAIR scans corrected for bias fields using the n4ITK filter. WMH were segmented manually with a minimum WMH intensity threshold, set at the intensity of the most intense voxels in the insular cortex, where cortical intensity was generally the highest. This was done to optimize reproducibility and consistency between scans (Olsson et al. 2013). This process was repeated for 10 random scans to test for intra-rater reliability. WMH maps were normalized to the NbM atlas and then used to calculate patients’ total WMH volume and WMH volume overlapping with the NbM pathway. We operationalized cerebral atrophy as 1 minus the ratio of total brain volume to intra cranial volume. Intra cranial and total brain volumes were obtained using Statistical Parametric Mapping (SPM) 8 on individual T1 scans (Ashburner 2012). All calculations were performed using Functional Software Library (FSL) software (Jenkinson et al. 2012; Woolrich et al. 2009).

### Statistical analysis

Statistical analyses were performed using SPSS 21 (IBM corp., Armonk, NY). Intra-rater reproducibility was assessed using the Sorensen-Dice Similarity Index: twice the volume of overlap (Vo) between two reconstructions divided by the total volume (V1 + V2) of those reconstructions (2*Vo/(V1 + V2)) (Sørensen 1948; Dice 1945). Differences between groups were assessed using student t-, chi square and Mann-Whitney U tests, where appropriate. To best fit the distribution of the number of symptoms per patient, Poisson linear regression was used to analyze the relation between mean FA and MD values and the total number of symptoms of the CDS. Binary logistic regression was used to assess the relation between mean FA and MD and the presence of CDS, defined as >1 CDS symptom. For comparison, the same models were used to assess the relation between mean FA and MD and the *non*-CDS symptoms. Models with FA based predictors were adjusted for cerebral atrophy and overall mean FA. Models with MD based predictors were adjusted for cerebral atrophy and overall mean MD. Poisson linear regression and binary logistic regression were also used to assess the association of WMH overlap with the number of CDS symptoms and the presence of the CDS respectively. In an exploratory setting, the mortality hazard was assessed using Cox proportional hazard models with standardized FA or MD as predictor, adjusted for cerebral atrophy and respectively for overall FA or MD. Also, subgroup analyses were performed regarding number of CDS symptoms in patients with and without a diagnosis of dementia. In addition, analyses were performed in which age, MMSE-score and use of medication potentially interfering with the frequency of NPI neuropsychiatric symptoms were also included in the adjusted model, next to cerebral atrophy and overall FA or MD. Potentially interfering medication comprised anticholinergics, antidepressants, neuroleptics, and any medication documented to cause any of the NPI neuropsychiatric symptoms in ≥1 % of users. Finally, exploratory analyses were performed assessing the relation between mean NbM FA and MD values and cerebral atrophy and MMSE score. These analyses were restricted to the largest diagnostic subgroup, patients with Alzheimer’s disease, since results would likely be confounded by the diagnosis.

## Results

### Clinical characteristics

Between March 2010 and August 2012, 110 patients with cognitive complaints were screened for eligibility. For 91 patients diffusion weighted imaging was available and of sufficient quality for tractography. A complete examination including the NPI was available for 87 of these patients, fulfilling our inclusion criteria. Screened patients with incomplete data (*n* = 23) did not differ significantly from included patients (Supplementary Table 1). Of the 87 included patients, 39 (45 %) were at the time of assessment diagnosed with possible or probable Alzheimer’s disease, 6 (7 %) with probable vascular dementia, 6 (7 %) with another type of dementia (4 frontotemporal dementia, 1 Parkinson’s disease dementia and 1 cortico basal degeneration), 26 (30 %) with mild cognitive impairment, and 10 (12 %) with subjective cognitive complaints. Survival data were available for 86 patients, one patient lost to follow-up due to emigration. Median follow-up time was 36 months (IQR: 34–36). During follow-up 13 patients died (median survival time: 21 months, IQR: 13–30). An overview of the neuropsychological test results per diagnostic category is provided in Supplementary Table 2.

Patient characteristics and a summary of the NPI results are listed for the patients with CDS (*n* = 29) versus those without in Table 1. Of the 29 CDS patients, 17 (59 %) were diagnosed with possible or probable Alzheimer’s disease, 7 (24 %) with mild cognitive impairment, 1 (3 %) with vascular dementia, 1 (3 %) with subjective cognitive complaints and 3 (10 %) with another type of dementia. Patients with CDS had a significantly higher NPI score than patients without CDS (median: 27, IQR: 24–42 vs. median: 3.5, IQR: 0–9; *p* < 0.001), and more *non*-CDS symptoms on the NPI (median 4, IQR: 3–5 vs. median 0, IQR: 0–1, p < 0.001). There were no significant differences regarding age, gender, MMSE score, acetylcholinesterase inhibitor use, cerebral atrophy and WMH volume.

**Table 1:** General characteristics for the whole study population (Total), patients with cholinergic deficiency syndrome (CDS) defined as >1 CDS symptom and patients without CDS. Symptoms were measured using the 12-symptom Neuropsychiatric Inventory (NPI). CDS symptoms comprised agitation, anxiety, apathy, delusions, hallucinations and irritability

Characteristic	Total (*n* = 87)	CDS (n = 29)	No CDS (*n* = 58)
Age (IQR)	79 (71–83)	80 (74–86)	78 (70–83)
Female, n (%)	46 (53 %)	17 (59 %)	29 (50 %)
MMSE Score, m (IQR)	25 (22–27)	25 (22–26)	25 (23–27)
Acetylcholinesterase inhibitor use	2 (2 %)	1 (3 %)	1 (2 %)
NPI score	9 (1–24)	27 (24–42)	(0–9)**
# NPI symptoms, m (IQR)	1 (0–3)	4 (3–5)	0 (0–1)**
> 1 CDS symptom, n (%)	29 (33 %)	29 (100 %)	0 (0 %)**
# CDS symptoms, m (IQR)	0 (0–2)	2 (2–3)	0 (0–0)**
> 1 non-CDS symptom, n (%)	15 (17 %)	12 (41 %)	3 (5 %)**
# non-CDS symptoms, m (IQR)	0 (0–1)	1 (1–2)	0 (0–1)**
Cerebral atrophy	45 % (5 %)	45 % (6 %)	44 % (4 %)
WMH volume (ml)	9.9 (5.7–15.9)	11.1 (7.9–14.9)	9.2 (4.9–16.0)

Means and standard deviations unless stated otherwise. Asterisks denote significant differences of CDS vs no CDS

*WMH* white matter hyperintensity

*p<0.05, **p<0.001

### NbM cortical pathway reconstruction and WMH segmentation

**Fig. 2:**
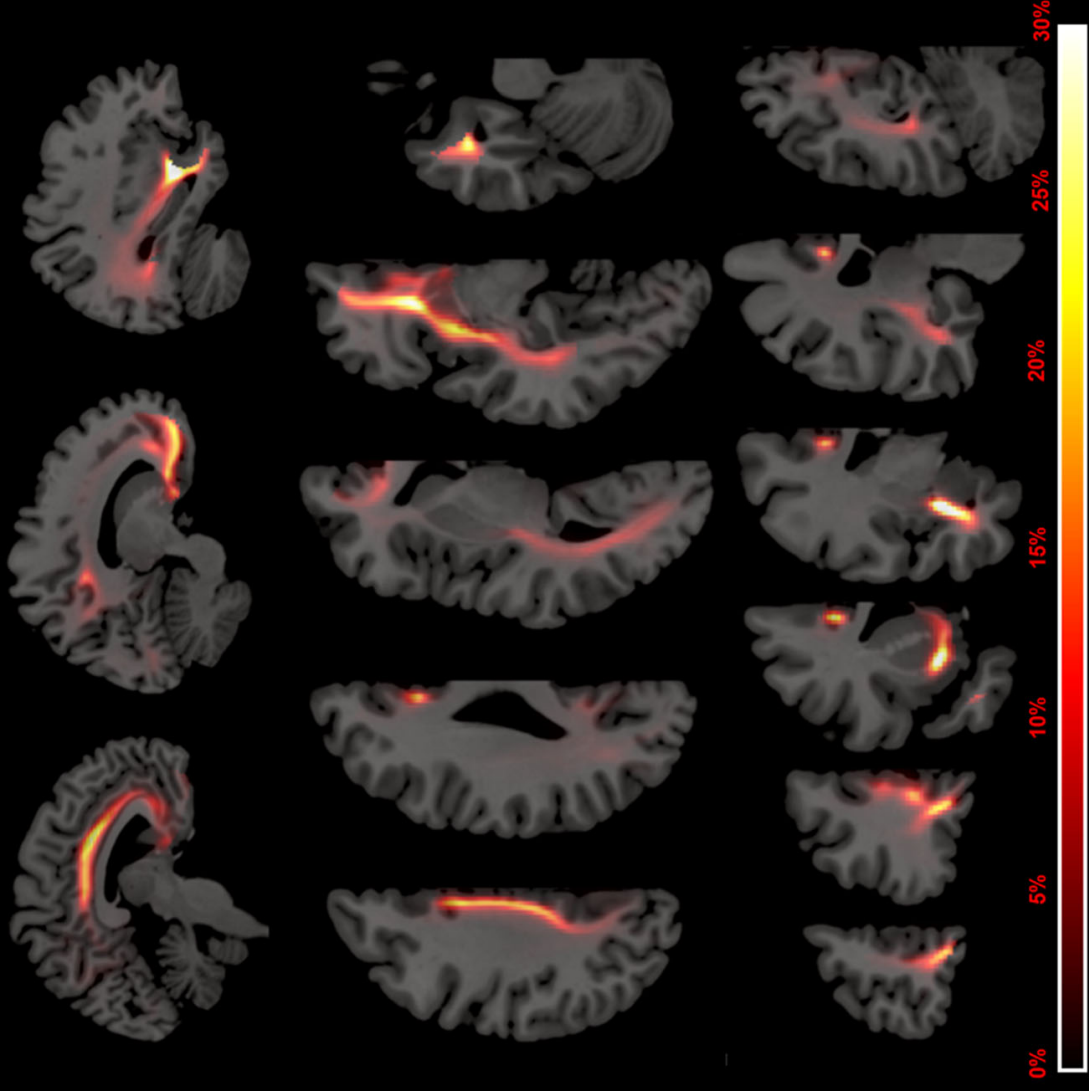
Tracking results. Combined population atlas of the nucleus basalis of Meynert (NbM) cortical pathway, projected over a normal space T1 scan. Colors represent probability map intensity ranging from 0 to 30 %. The mean fractional anisotropy and mean diffusivity was analysed in the regions overlapping with the atlas thresholded at 5 %. *Top row:* sagittal slices from medial (left) to lateral (right). *Middle row*: axial slices from dorsal (left) to ventral (right). *Bottom row*: coronal slices from anterior (left) to posterior (right)

**Fig. 3:**
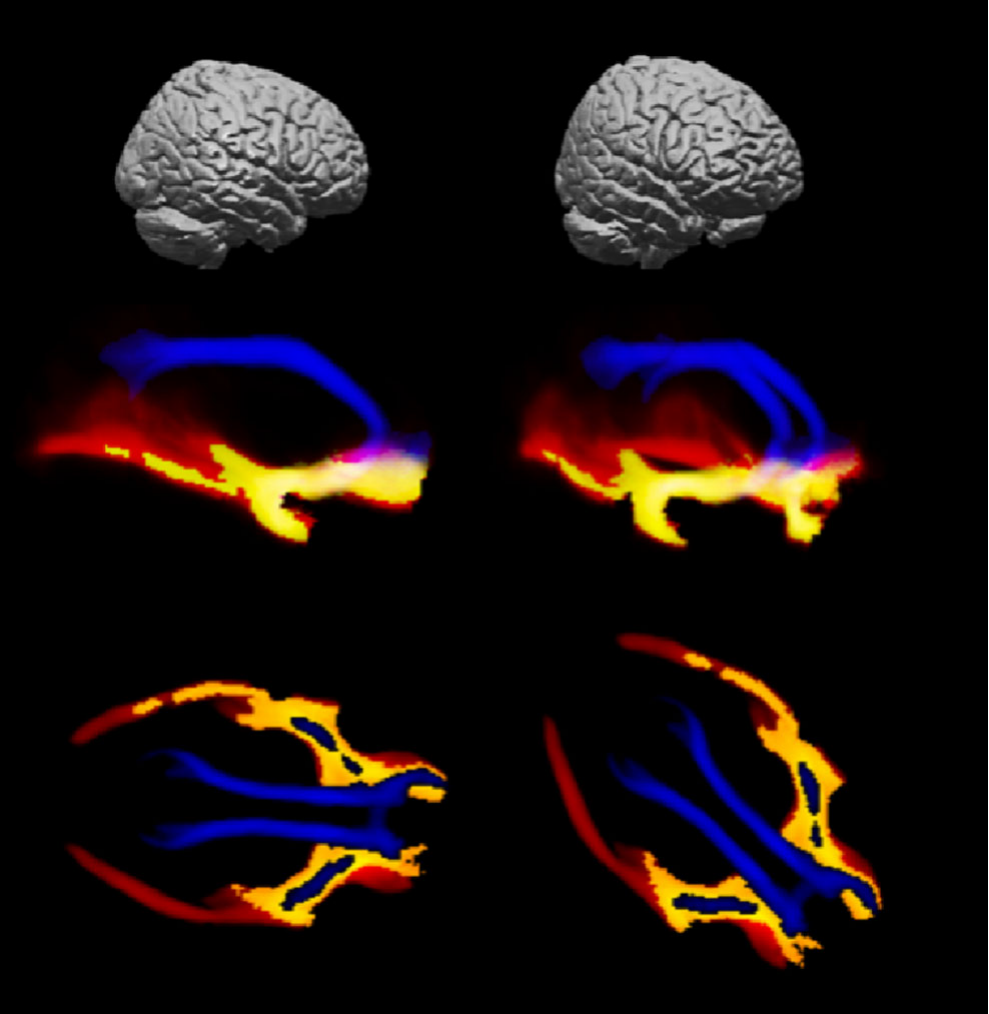
Tracking results. 3D view of the nucleus basalis of Meynert (NbM) cortical pathway reconstructions thresholded at 5 % with colours depicting the contribution of the projections from the medial and lateral seed ROI separately. Blue: contribution of projections from the medial seed ROI. Red: contribution of projections from the lateral seed ROI. Yellow: overlap between the projections from the medial and lateral seed ROIs. *Top row:* 3D brain depicting the viewing angle of the middle row (not to scale). *Middle row:* lateral view of the 3D reconstruction at an angle of 90, 45 and 20 degrees from the frontal view. *Bottom row:* craniocaudal view of the 3D reconstruction depicted in the middle row

Tracking results proved reproducible with an average similarity index of 0.84. Reproducibility of WMH segmentation was also good with an average similarity index of 0.86. The atlas generated by combining the tracking results of all patients is depicted in Fig. 2. A 3D reconstruction of the atlas with color-coding of the separate reconstructions from the lateral and medial ROIs and their overlap is shown in Fig. 3. The proximal, intermediate and distal divisions of the NbM cortical pathway are shown in Supplementary Fig. 1. A standardized version of the atlas is available to view and download online.

### CDS symptoms and cholinergic projections

Results of analyses of CDS symptoms in relation to of FA and MD in the NbM cortical pathway are listed in Table 2. Lower FA values and higher MD values in the NbM cortical pathway were associated with more CDS symptoms, specifically in the proximal and the intermediate parts. These associations remained significant when adjusted for cerebral atrophy and overall FA or MD. Lower overall FA was not associated with more CDS symptoms.

**Table 2:** Results of Poisson linear regression of total number of cholinergic deficiency syndrome (CDS) symptoms predicted by the fractional anisotropy (FA) and mean diffusivity (MD) in the listed regions, and binary logistic regression of >1 symptom of the CDS predicted by the fractional anisotropy (FA) and mean diffusivity (MD) in the listed regions. NbM: total nucleus basalis of Meynert (NbM) cortical pathway, Proximal: proximal part only, Intermediate: intermediate part only, Distal: distal part only. NbM medial and lateral ROI projections: cortical pathway from the lateral and medial seed ROI separately

		number of CDS symptoms							
		model 1 (unadjusted)				model 2 (adjusted)*			
		beta	95 % CI		p	beta	95 % CI		P
FA	NbM	-0.24	-0.45	-0.03	0.02	-0.22	-0.44	0.00	0.05
	- Proximal	-0.26	-0.46	-0.06	0.01	-0.24	-0.45	-0.03	0.03
	- Intermediate	-0.27	-0.46	-0.07	0.01	-0.28	-0.48	-0.07	0.01
	- Distal	-0.15	-0.36	0.05	0.14	-0.14	-0.37	0.09	0.22
	NbM medial ROI projections	-0.21	-0.40	-0.13	0.04	-0.21	-0.43	0.00	0.05
	NbM lateral ROI projections	-0.24	-0.44	-0.04	0.02	-0.23	-0.45	-0.02	0.03
	Whole brain (reference)	0.09	-0.12	0.30	0.39	0.00	-0.24	0.24	0.99
MD	NbM	0.34	0.14	0.53	0.00	0.32	0.01	0.63	0.05
	- Proximal	0.29	0.12	0.46	0.00	0.28	0.01	0.55	0.04
	- Intermediate	0.35	0.16	0.53	0.00	0.30	0.06	0.55	0.02
	- Distal	0.23	0.05	0.41	0.01	0.16	-0.05	0.38	0.14
	NbM medial ROI projections	0.23	0.08	0.38	0.00	0.23	0.02	0.43	0.03
	NbM lateral ROI projections	0.33	0.14	0.52	0.00	0.32	0.02	0.62	0.04
	Whole brain (reference)	0.20	0.03	0.37	0.02	0.21	0.04	0.38	0.02
		> 1 symptom of CDS							
		model 1 (unadjusted)				model 2 (adjusted)*			
		OR	95 % CI		p	OR	95 % CI		P
FA	NbM	0.63	0.39	1.01	0.06	0.63	0.38	1.03	0.06
	-Proximal	0.62	0.39	0.99	0.05	0.62	0.38	1.00	0.05
	-Intermediate	0.55	0.33	0.92	0.02	0.55	0.33	0.92	0.02
	-Distal	0.75	0.47	1.19	0.22	0.75	0.46	1.20	0.22
	NbM medial ROI projections	0.64	0.39	1.05	0.08	0.64	0.39	1.04	0.07
	NbM lateral ROI projections	0.60	0.37	0.96	0.04	0.63	0.39	1.02	0.06
	Whole brain (reference)	1.04	0.67	1.63	0.85	0.95	0.56	1.62	0.85
MD	NbM	1.79	1.10	2.91	0.02	2.05	0.96	4.38	0.06
	-Proximal	1.86	1.13	3.05	0.02	2.17	1.03	4.55	0.04
	-Intermediate	1.82	1.13	2.93	0.01	1.84	0.99	3.43	0.05
	-Distal	1.56	0.97	2.51	0.07	1.41	0.84	2.39	0.20
	NbM medial ROI projections	1.72	1.01	2.93	0.05	1.78	0.95	3.32	0.07
	NbM lateral ROI projections	1.78	1.10	2.88	0.02	1.98	0.95	4.14	0.07
	Whole brain (reference)	1.39	0.88	2.19	0.16	1.4	0.89	2.22	0.15

*Corrected for cerebral atrophy and whole brain FA for FA based predictors and whole brain MD for MD based predictors

Higher overall MD was associated with more CDS symptoms but less strongly than MD in the NbM cortical pathway. Analyses with >1 symptom of CDS as outcome yielded similar results, with slightly weaker correlations. As a control condition, the same analyses with the number of *non*-CDS symptoms and >1 *non*-CDS symptom as outcomes did not reveal any significant associations (Supplementary Table 3 I-II).

Results of analyses regarding CDS symptoms and FA and MD for the reconstructions from the medial and lateral seed ROI separately were similar (Table 2). The association of CDS symptoms with lower FA and higher MD were found in both the lateral and the medial seed ROI based reconstructions.

Exploratory analyses for the number of CDS symptoms in dementia diagnosis subgroups showed results similar to those of the main analyses, as did exploratory analyses with additional adjustment for age, sex and medication potentially interfering with the number of CDS symptoms (Supplementary Table 4 I-III). In the exploratory analyses regarding cerebral atrophy and MMSE score, we found a strong relation of lower FA and higher MD values in the NbM and greater cerebral atrophy in Alzheimer’s disease patients, not present for overall FA and MD (Supplementary Table 4 IV). We found no relation between FA and MD values and MMSE score in patients with Alzheimer’s disease (Supplementary Table 4 V).

**Table 3.** Results of Poisson linear regression of total number of cholinergic deficiency syndrome (CDS) symptoms predicted by the overlap between white matter hyperintensities (WMH) and the listed regions, and binary logistic regression of the presence of >1 symptom of the cholinergic deficiency syndrome (CDS) predicted by the overlap between white matter hyperintensities (WMH) and the listed regions

		number of CDS symptoms			
		beta	95 % CI		p
WMH	NbM overlap volume	0.09	-0.10	0.27	0.36
	-Proximal	0.12	-0.06	0.29	0.19
	-Intermediate	0.07	-0.11	0.25	0.46
	-Distal	0.05	-0.14	0.25	0.59
	Total volume (reference)	0.08	-0.11	0.26	0.41
		>1 symptom of CDS			
		OR	95 % CI		p
WMH	NbM overlap volume	0.81	0.40	1.66	0.57
	-Proximal	0.86	0.45	1.65	0.65
	-Intermediate	0.68	0.24	1.94	0.47
	-Distal	0.87	0.47	1.61	0.67
	Total volume (reference)	0.76	0.34	1.69	0.50

*NbM* total nucleus basalis of Meynert (NbM) cortical pathway, *Proximal* proximal part only, *Intermediate* intermediate part only, *Distal* distal part only, *Total volume* total brain WMH volume

### CDS symptoms and WMH

Results of regression analyses assessing the relation between the number of CDS symptoms and the amount of WMH overlap with the NbM-cortical pathway are listed in Table 3. We found no association between WMH volume in the NbM cortical pathway and the number of CDS symptoms. There was also no association between total WMH volume and the number of CDS symptoms. Results of the binary logistic analyses with the same predictors and the presence of the CDS were consistent.

### NbM cortical pathway degradation and mortality

**Fig. 4:**
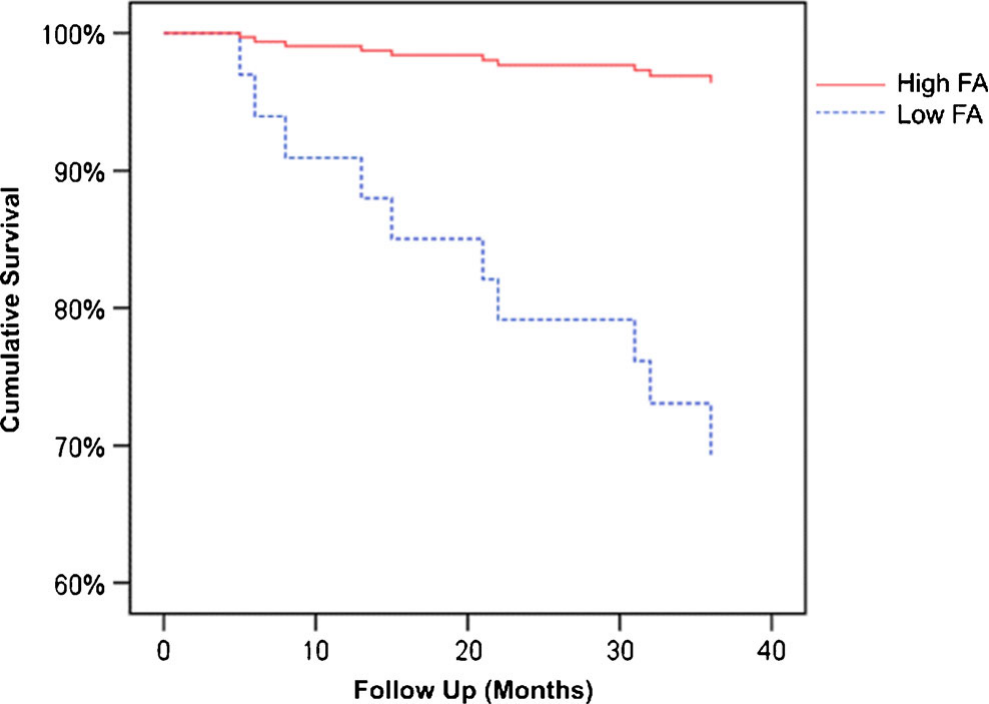
Survival curve high vs. low fractional anisotropy. Patients dichotomized according to high and low mean fractional anisotropy (FA) values in the proximal part of the nucleus basalis of Meynert (NbM) cortical pathway. For mortality, one standard deviation higher mean FA in the proximal NbM cortical pathway was associated with a hazard ratio of 0.57 (95%CI: 0.34–0.94) (Table 5)

Results of Cox proportional hazard analyses with the standardized FA and MD are shown in Supplementary Table 4 VI. Lower FA in the NbM cortical pathway, particularly in the proximal and distal segments, was associated with a two-third higher mortality hazard per standard deviation, in contrast to overall FA. After adjustment for cerebral atrophy and overall brain FA, these hazard ratios were no longer significant although the effect sizes remained similar. The association between the FA in the proximal NbM cortical pathway and increased mortality is further illustrated in Fig. 4. Higher MD in the NbM cortical pathway was also associated with higher mortality, although hazard ratios were comparable to those of the overall MD. Consequently, these associations disappeared when adjusted for cerebral atrophy and overall MD.

## Discussion

Our study shows that degradation of the NbM cortical projection pathway, as indicated by lower mean FA and higher MD values, is significantly correlated with clinical symptoms of cholinergic deficiency in patients presenting to a memory clinic. These CDS symptoms were represented by NPI domains for agitation, anxiety, apathy, delusions, hallucinations, and irritability, and considered present when exceeding a frequency by severity domain score > 3. The relation between CDS symptoms and NbM degeneration is independent of cerebral atrophy and whole brain FA and MD values. The correlation is particularly strong in the regions proximal to the NbM. The association is specific to symptoms of the CDS compared to other neuropsychiatric symptoms and specific to degradation of this pathway compared to overall structural degradation in the brain. We did not find any association of CDS symptoms with WMH volume in the NbM cortical pathway, nor with WMH volume in general.

Our findings are consistent with the hypothesis that disruptions of the cholinergic NbM projections may evoke cortical cholinergic deficiency and CDS symptoms (Lemstra et al. 2003; Pinto et al. 2011). However, we cannot discern whether the CDS can be attributed specifically to degradation of the cholinergic NbM projections, since tractography cannot differentiate between these and other non-cholinergic projections in the same pathway. These non-cholinergic projections largely originate from the midbrain monoamine neuron nuclei, posterior hypothalamus and basal forebrain, and make up the majority of the pathway between the NbM and the cortex (Saper et al. 2010; 2005). Projections in this pathway are involved in regulating wakefulness, motivation, reward seeking, and related functions (Saper et al. 2010; Mahler et al. 2014; Larson-Prior et al. 2014). Arguably, dysfunction of many of these projections could induce neuropsychiatric symptoms, including those of the CDS. However, other findings relate CDS symptoms to deficiency of the cholinergic NbM projections. CDS symptoms are particularly susceptible to cholinergic treatment (Lemstra et al. 2003; Pinto et al. 2011). The fundamental CDS features, disturbed attention and stimulus processing, have been linked to deficiencies in cortical cholinergic stimulation (Lemstra et al. 2003; Pinto et al. 2011; Saper et al. 2010; Gratwicke et al. 2013), which is derived almost exclusively from the NbM (Mesulam 2013). Finally, degradation of the NbM has been linked to disturbances in attention and stimulus processing (Lemstra et al. 2003; Pinto et al. 2011; Gratwicke et al. 2013). This suggests CDS symptoms in particular are associated with degradation of the cholinergic NbM projections.

That proximal disruptions of the NbM projections have more impact on cortical cholinergic activity concurs with previous findings (Teipel et al. 2011; Bocti et al. 2005; Bohnen et al. 2009). Possibly, these have a more general effect, by disrupting cholinergic fibers before they spread throughout the brain more distally (Teipel et al. 2011; Selden et al. 1998; Bohnen et al. 2009). Alternatively, as the distal projections fan out (Selden et al. 1998), inter individual anatomical variation may become too large to accurately capture in an atlas model.

In addition to the cross-sectional association with neuropsychiatric symptoms, our results suggest that degradation of the NbM cortical projections may be associated with an increased risk of mortality during three year follow-up. This might mean that NbM degeneration is associated with a worse prognosis and possibly a marker of more advanced disease. However, the relation between NbM projection integrity and mortality in our study was only a trend, since it was not significant after adjustment, and was based on exploratory analyses in a relatively small study population with a limited number of events.

Our results do not support our hypothesis that structural damage to the NbM projections by WMH leads to CDS symptoms. Other studies have associated WMH in general with neuropsychiatric symptoms (Berlow et al. 2010; Kee Hyung Park et al. 2011). Furthermore, WMH in the proximal NbM projections have been linked to worse executive functioning and increased dementia severity, supposedly through cholinergic impairment (Bocti et al. 2005; Bohnen et al. 2009; Behl et al. 2007). Finally, lesions within the NbM projections have been associated with visuospatial attention disorders, which play an important role in the CDS (Lemstra et al. 2003; Swartz et al. 2003). As an explanation, the relation between WMH and symptoms of cholinergic deficiency may be specific to the type of dementia. Cortical cholinergic deficiency in vascular dementia may depend on strategic lesions in the NbM cholinergic projections, although many Alzheimer’s disease patients also have cerebrovascular pathology, the effect of lesions in the NbM projections may be relatively small since NbM function is already impaired by primary degeneration of the NbM, which occurs from the earliest stages in Alzheimer’s disease (Kim et al. 2013, Grothe et al. 2012). The majority of dementia patients in our study population have Alzheimer’s disease. The number of vascular dementia patients in our sample was too small to test this hypothesis. Also, methodological differences may play a role, other studies most often employing visual rating scales to assess the impact of WMH in the NbM projections (Bocti et al. 2005; Bohnen et al. 2009; Fukui et al. 2006; Kim et al. 2012; Behl et al. 2007). Finally, although morphological studies have shown that the cholinergic fibers within WMH are often severed (Roman and Kalaria 2006), it is possible that WMH do not functionally disrupt cholinergic fiber integrity enough to impact cholinergic innervation.

This study has some limitations. Although we defined the CDS symptoms based on previously proposed clinical characteristics (Lemstra et al. 2003; Pinto et al. 2011), no formal clinical criteria exist. The CDS and its composite symptoms are still a putative concept and need validation. Our operationalization of the CDS as having more than 1 CDS symptoms was arbitrary, we therefore made the number of CDS symptoms the main focus in our interpretations of the analyses. The total number of CDS cases was relatively small (*n* = 29), limiting the number of covariates in our regression models. This limitation is stronger in the comparison with patients with >1 *non*-CDS symptom (*n* = 15), possibly allowing for a type II error. Similarly, our study population may have been too small to detect a relation between WMH volume and CDS symptoms. Relatively small study size may also have affected our exploratory survival analyses, which were based on 13 events for the whole population. Finally, the small study size undermined our capacity to perform exploratory analyses regarding to which degree individual neuropsychiatric symptoms were associated with loss of integrity to the projections of the NbM. Our study is focused on a sample with almost no use of acetylcholinesterase inhibitors (only 2 participants), which left us unable to investigate their effect on the relation between NbM pathway degeneration and the CDS. By restricting our analyses to a literature based predefined cluster of symptoms we aimed to avoid spurious results. However, the limitation of this approach is that our operationalization of CDS symptoms may be incomplete or include symptoms which are not mediated by dysfunction of the cholinergic projections from the NbM. Next to small in size, the study population is also heterogeneous in age, medication use and diagnoses underlying participants’ cognitive complaints. Exploratory analyses in patients with and without dementia and adjusted for age and medication use however gave similar results regarding the relation between NbM projection integrity and CDS symptoms. Regarding tracking methodology, it could be argued that combining reconstructions from two separate seed ROIs predominantly increases the number of false positive results. However, our reconstructions of the NbM pathways are consistent with morphological descriptions based on meticulous anatomical and neuropathological studies (Fig. 2) (Teipel et al. 2011; Selden et al. 1998). Since both seed ROIs contributed valid and unique parts to the overall reconstruction (Fig. 3), their combination optimized our tracking results. Another concern may be that the exact location of the seed ROI was not fully standardized but rather identified per patient. We employed this method to allow for the tailoring of the ROI to the likely NbM location, taking into account inter individual anatomical variation and distortion by regional cerebral atrophy (Zaborszky et al. 2008). The good reproducibility of the tracking results supports the use of this approach.

Our findings show that the clinically defined Cholinergic Deficiency Syndrome has a functional neuroanatomical correlate that can be assessed in vivo using structural MRI. They support the hypothesis that damage to the integrity of the cholinergic NbM projections may provoke the specific cluster CDS symptoms (Lemstra et al. 2003; Pinto et al. 2011). Our results are relevant for research into the role of the NbM projections in the development of cognitive and behavioral deficits. Other studies of brain-behavior relations can easily employ our developed atlas of the NbM cortical pathways. Studies in larger cohorts with longer follow-up may be able to substantiate the supposed trend for increased mortality with loss of NbM projection integrity. Others may use our atlas to study whether measures of NbM integrity could be useful for identification of memory clinic patients likely to benefit from cholinergic medication, NbM deep brain stimulation, or with a worse prognosis (Brousseau et al. 2007; Pinto et al. 2011; Grothe et al. 2010; Van Beek and Claassen 2011; Herholz et al. 2008). Our method of exploratory tractography may be helpful to researchers looking to identify projections from anatomically ill-defined regions, particularly those with large inter subject-variability or sensitive to distortion by cerebral atrophy. Finally, our results show how state-of-the-art neuroimaging techniques can be instrumental in uncovering brain-behavior relations, combining both clinical and neuroscientific relevance.

## Electronic supplementary material

1 (DOC 381 kb)

2 (ZIP 2.26 mb)

## Abbreviations

CDS: Cholinergic Deficiency Syndrome
NbM: Nucleus Basalis of Meynert
WMH: White Matter Hyperintensities of presumed vascular origin
FA: Fractional Anisotropy
MD: Mean Diffusivity
MMSE: Mini Mental Stage Examination
NPI: Neuropsychiatric Inventory
ROI: Region Of Interest
