# Supplementary material for: Neuropsychiatric symptoms of cholinergic deficiency occur with degradation of the projections from the nucleus basalis of Meynert
Source: Brain Imaging Behav. 2016 Oct 27;11(6):1707–19. doi: 10.1007/s11682-016-9631-5 (PMC5707238; doi:10.1007/s11682-016-9631-5)
Supplement: Supplementary file 1 — (DOC 381 kb) [file 11682_2016_9631_MOESM1_ESM.doc]

**Neuropsychiatric symptoms of cholinergic deficiency occur with degradation of the projections from the nucleus basalis of Meynert Supplementary material.**

Supplementary Figure 1: subdivisions of the nucleus basalis cortical pathways (page 2)
Supplementary Table 1: included versus excluded patients (page 2)
Supplementary Table 2: neuropsychological test battery scores
Supplementary Table 3 I-II: non-CDS reference tables (page 3)
Supplementary Table 4 I-VI: exploratory analyses (page 4)

**Supplementary Figure 1, subdivisions of the nucleus basalis cortical pathways**

**
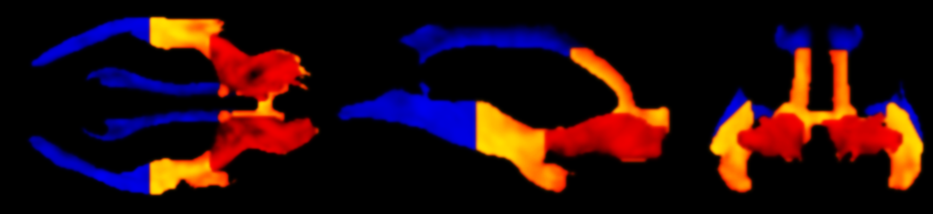
**

**Supplementary Figure 1. Proximal, intermediate and distal subdivisions.** From left to right: bottom-up, side and frontal view of 3D reconstruction of the nucleus basalis cortical pathways thresholded at 5% with colours depicting the proximal (red), intermediate (orange) and distal (blue) subdivisions of the tracking results.

**Supplementary Table 1, included vs. excluded patients**

| Characteristics | Included (n=87) | Not Included (n=23) | |
| --- | --- | --- | --- |
|  |  | overall | valid n (%) |
| Age, m (IQR) | 79 (67-81) | 76 (69-83) | 14 (61%) |
| Female, n (%) | 46 (53%) | 6 (43%) | 14 (61%) |
| MMSE Score, m (IQR) | 25 (22-27) | 25 (24-27) | 13 (57%) |
| SCC, n (%) | 10 (12%) | 1 (7%) | 14 (61%) |
| MCI, n (%) | 26 (30%) | 4 (29%) | 14 (61%) |
| Dementia, n (%) | 51 (59%) | 9 (64%) | 14 (61%) |
| Atrophy | 45 (5) | 45 (4) | 6 (26%) |
| WMH load | 4 (2-7) | 5 (3-18) | 6 (26%) |
| Overall FA | 19 (1.4) | 19 (1.4) | 6 (26%) |
| Overall MD | 55 (8.2) | 54 (7.2) | 6 (26%) |
| **Supplementary Table 1.** General characteristics of patients included in the analysis vs. those who were not included. Percentages were based upon the total number of patients for whom data was available. Data was complete for included patients, the number of not included patients for whom data was available is listed in the “valid n (%)” column. MMSE: mini mental state examination, SCC: subjective cognitive complaints, MCI: mild cognitive impairment, WMH load: white matter hyperintensity volume per total brain volume (*10^5^), Overall FA: fractional anisotropy (*10^4^), Overall MD: mean diffusivity (*10^5^). Means and standard deviations unless stated otherwise. | | | |

**Supplementary Table 2: neuropsychological test battery scores:**

| Diagnostic subgroup |  | Memory and Learning | | | | | | | | | | | | Verbal fluency | | |
| --- | --- | --- | --- | --- | --- | --- | --- | --- | --- | --- | --- | --- | --- | --- | --- | --- |
|  |  | VAT | | | 15-WLT recall | | | 15-WLT delayed recall | | | Digit-Span | | | Animal Fluency | | |
|  | n | n (%) | mean | SD | n (%) | mean | SD | n | mean | SD | n (%) | mean | SD | n (%) | mean | SD |
| Subjective cognitive complaints | 10 | 5 (50%) | 20 | 11 | 5 (50%) | 36 | 13 | 5 (50%) | 38 | 8 | 5 (50%) | 55 | 11 | 5 (50%) | 45 | 7 |
| Mild cognitive impairment | 26 | 18 (69%) | 15 | 12 | 18 (69%) | 29 | 8 | 18 (69%) | 26 | 14 | 17 (65%) | 48 | 9 | 18 (69%) | 39 | 8 |
| Alzheimer’s disease | 39 | 26 (67%) | 5 | 8 | 25 (64%) | 25 | 11 | 24 (62%) | 18 | 15 | 26 (67%) | 48 | 7 | 25 (64%) | 37 | 12 |
| Other | 12 | 9 (75%) | 14 | 12 | 9 (75%) | 29 | 9 | 9 (75%) | 29 | 9 | 9 (75%) | 48 | 9 | 9 (75%) | 35 | 10 |

**Supplementary Table 2 I:** Means and standard deviations (SD) of neuropsychological test results per diagnostic subgroup normalized to general population data adjusted for sex, age and education. n (%): number of valid measurements and percentage of total, Other: includes Lewy body dementia, fronto-temporal dementia, coritico-basal degeneration and Parkinson’s disease dementia, no neuropsychological test scores were available for patients with vascular dementia (n=6), VAT: visual association test, 15 WLT recall: 15-word learning test, Digit-Span: WAIS III Digit-Span (forward and backward).

| Diagnostic subgroup |  | Information processing speed and executive functioning | | | | | | | | | | | | | | |
| --- | --- | --- | --- | --- | --- | --- | --- | --- | --- | --- | --- | --- | --- | --- | --- | --- |
|  |  | Stroop Test | | | Trail Making Test A | | | Trail Making Test B | | | LDST written | | | LDST verbal | | |
|  | n | n (%) | mean | SD | n (%) | mean | SD | n | mean | SD | n (%) | mean | SD | n (%) | mean | SD |
| Subjective cognitive complaints | 10 | 5 (50%) | 39 | 7 | 5 (50%) | 38 | 21 | 5 (50%) | 37 | 23 | 5 (50%) | 36 | 7 | 5 (50%) | 40 | 10 |
| Mild cognitive impairment | 26 | 18 (69%) | 43 | 14 | 18 (69%) | 46 | 13 | 17 (65%) | 39 | 19 | 17 (65%) | 35 | 21 | 18 (69%) | 35 | 19 |
| Alzheimer’s disease | 39 | 24 (62%) | 36 | 17 | 25 (64%) | 38 | 17 | 24 (62%) | 27 | 20 | 25 (64%) | 32 | 12 | 23 (59%) | 32 | 12 |
| Other | 12 | 9 (75%) | 33 | 10 | 9 (75%) | 33 | 15 | 7 (58%) | 34 | 18 | 9 (75%) | 29 | 19 | 8 (67%) | 27 | 19 |

**Supplementary Table 2 II:** Means and standard deviations (SD) of neuropsychological test results per diagnostic subgroup normalized to general population data adjusted for sex, age and education. n (%): number of valid measurements and percentage of total, Other: includes Lewy body dementia, fronto-temporal dementia, coritico-basal degeneration and Parkinson’s disease dementia, no neuropsychological test scores were available for patients with vascular dementia (n=6), Stroop Test: part 3, LDST: letter digit substitution test.

**Supplementary Table 3 I-II: non-CDS reference tables**

|  |  | number of non-CDS symptoms | | | | > 1 non-CDS symptom | | | |
| --- | --- | --- | --- | --- | --- | --- | --- | --- | --- |
|  |  | beta | 95% CI | | p | OR | 95% CI | | p |
| FA | NbM | -0.11 | -0.35 | 0.14 | 0.39 | 0.83 | 0.47 | 1.47 | 0.53 |
|  | - Proximal | -0.11 | -0.35 | 0.13 | 0.38 | 0.84 | 0.48 | 1.46 | 0.54 |
|  | - Intermediate | -0.13 | -0.37 | 0.10 | 0.26 | 0.84 | 0.49 | 1.46 | 0.55 |
|  | - Distal | -0.07 | -0.31 | 0.17 | 0.58 | 0.92 | 0.53 | 1.60 | 0.76 |
|  | NbM medial ROI projections | -0.10 | -0.34 | 0.13 | 0.39 | 0.90 | 0.52 | 1.56 | 0.70 |
|  | NbM lateral ROI projections | -0.10 | -0.34 | 0.14 | 0.41 | 0.85 | 0.49 | 1.48 | 0.57 |
|  | Whole brain (reference) | 0.20 | -0.04 | 0.44 | 0.10 | 1.09 | 0.62 | 1.90 | 0.77 |
| MD | NbM | 0.10 | -0.13 | 0.34 | 0.40 | 1.00 | 0.57 | 1.75 | 1.00 |
|  | - Proximal | 0.18 | -0.04 | 0.39 | 0.11 | 1.15 | 0.68 | 1.96 | 0.60 |
|  | - Intermediate | 0.22 | -0.01 | 0.44 | 0.06 | 1.21 | 0.70 | 2.07 | 0.49 |
|  | - Distal | 0.09 | -0.14 | 0.32 | 0.44 | 0.93 | 0.52 | 1.66 | 0.81 |
|  | NbM medial ROI projections | 0.13 | -0.07 | 0.34 | 0.20 | 1.03 | 0.60 | 1.78 | 0.91 |
|  | NbM lateral ROI projections | 0.18 | -0.04 | 0.41 | 0.11 | 1.15 | 0.66 | 1.98 | 0.62 |
|  | Whole brain (reference) | -0.04 | -0.29 | 0.21 | 0.74 | 0.95 | 0.53 | 1.69 | 0.86 |
| **Supplementary Table 3 I**. Poisson linear regression of the total number of symptoms on the neuropsychiatric inventory (NPI) not belonging to the cholinergic deficiency syndrome (non-CDS symptoms) predicted by the fractional anisotropy (FA) and mean diffusivity (MD) in the listed regions and binary logistic regression of >1 non-CDS symptom. NbM: total nucleus basalis of Meynert cortical pathway, Proximal: proximal part only, Intermediate: intermediate part only, Distal: distal part only, NbM medial and lateral ROI projections: cortical pathway from the lateral and medial seed ROI separately. | | | | | | | | | |

|  |  | number of non-CDS symptoms | | | | >1 non-CDS symptom | | | |
| --- | --- | --- | --- | --- | --- | --- | --- | --- | --- |
|  |  | OR | 95% CI | | p | OR | 95% CI | | p |
| WMH | NbM overlap volume | 0.81 | 0.40 | 1.66 | 0.57 | -0.02 | -0.27 | 0.23 | 0.88 |
|  | - Proximal | 0.86 | 0.45 | 1.65 | 0.65 | 0.03 | -0.20 | 0.26 | 0.80 |
|  | - Intermediate | 0.68 | 0.24 | 1.94 | 0.47 | -0.09 | -0.38 | 0.20 | 0.55 |
|  | - Distal | 0.87 | 0.47 | 1.61 | 0.67 | -0.02 | -0.26 | 0.23 | 0.89 |
|  | Total volume (reference) | 0.76 | 0.34 | 1.69 | 0.50 | -0.07 | -0.34 | 0.20 | 0.62 |
| **Supplementary Table 3 II**. Poisson linear regression of the total number of symptoms on the neuropsychiatric inventory (NPI) not belonging to the cholinergic deficiency syndrome (non-CDS symptoms) and binary logistic regression of >1 non-CDS symptom, predicted by the overlap between white matter hyperintensities (WMH) and the listed regions. NbM: total nucleus basalis of Meynert cortical pathway, Proximal: proximal part only, Intermediate: intermediate part only, Distal: distal part only, Total volume: total brain WMH volume | | | | | | | | | |

**Supplementary Table 4 I-III: exploratory analyses:**

| Subgroup |  |  | Number of CDS symptoms | | | | | | | | | | | |
| --- | --- | --- | --- | --- | --- | --- | --- | --- | --- | --- | --- | --- | --- | --- |
|  |  |  | model 1 (unadjusted) | | | | | | model 2 (adjusted) * | | | | | |
|  |  |  | beta | | 95% CI | | p | | beta | | 95% CI | | P | |
| Patients without dementia (n=31) | FA | NbM | -0.20 | -0.70 | | 0.31 | | 0.44 | -0.09 | -0.62 | | 0.44 | | 0.74 |
|  |  | - Proximal | -0.12 | -0.61 | | 0.38 | | 0.64 | 0.02 | -0.48 | | 0.53 | | 0.93 |
|  |  | - Intermediate | -0.29 | -0.64 | | 0.05 | | 0.10 | -0.19 | -0.66 | | 0.28 | | 0.42 |
|  |  | - Distal | -0.19 | -0.60 | | 0.21 | | 0.35 | -0.04 | -0.50 | | 0.43 | | 0.88 |
|  |  | Whole brain (reference) | -0.09 | -0.59 | | 0.41 | | 0.73 | 0.22 | -0.40 | | 0.84 | | 0.48 |
|  | MD | NbM | 0.30 | -0.16 | | 0.75 | | 0.20 | -0.03 | -0.99 | | 0.93 | | 0.95 |
|  |  | - Proximal | 0.29 | -0.08 | | 0.65 | | 0.12 | -0.02 | -0.85 | | 0.82 | | 0.97 |
|  |  | - Intermediate | 0.36 | -0.03 | | 0.76 | | 0.07 | 0.28 | -0.47 | | 1.02 | | 0.47 |
|  |  | - Distal | 0.13 | -0.23 | | 0.48 | | 0.49 | -0.43 | -1.14 | | 0.27 | | 0.23 |
|  |  | Whole brain (reference) | 0.39 | -0.05 | | 0.83 | | 0.08 | 0.29 | -0.35 | | 0.93 | | 0.38 |
| Patients with dementia (n=51) | FA | NbM | -0.23 | -0.46 | | 0.00 | | 0.05 | -0.11 | -0.37 | | 0.15 | | 0.40 |
|  |  | - Proximal | -0.28 | -0.50 | | -0.06 | | 0.01 | -0.17 | -0.42 | | 0.08 | | 0.17 |
|  |  | - Intermediate | -0.25 | -0.50 | | -0.01 | | 0.04 | -0.12 | -0.40 | | 0.16 | | 0.40 |
|  |  | - Distal | -0.14 | -0.38 | | 0.10 | | 0.24 | -0.01 | -0.28 | | 0.27 | | 0.97 |
|  |  | Whole brain (reference) | 0.10 | -0.13 | | 0.32 | | 0.42 | -0.04 | -0.28 | | 0.19 | | 0.72 |
|  | MD | NbM | 0.32 | 0.10 | | 0.54 | | 0.00 | 0.22 | -0.15 | | 0.60 | | 0.24 |
|  |  | - Proximal | 0.28 | 0.08 | | 0.48 | | 0.01 | 0.16 | -0.18 | | 0.51 | | 0.36 |
|  |  | - Intermediate | 0.30 | 0.09 | | 0.52 | | 0.01 | 0.15 | -0.16 | | 0.45 | | 0.36 |
|  |  | - Distal | 0.32 | 0.07 | | 0.56 | | 0.01 | 0.12 | -0.21 | | 0.44 | | 0.48 |
|  |  | Whole brain (reference) | 0.15 | -0.03 | | 0.33 | | 0.11 | 0.12 | -0.07 | | 0.31 | | 0.22 |
| **Supplementary Table 4 I: dementia subgroup FA and MD analysis.** Poisson linear regression of total number of cholinergic deficiency syndrome (CDS) symptoms predicted by the fractional anisotropy (FA) and mean diffusivity (MD) in the listed regions separately for patients with and without a diagnosis of dementia. NbM: total nucleus basalis of Meynert cortical pathway, Proximal: proximal part only, Intermediate: intermediate part only, Distal: distal part only, *model 2: adjusted for atrophy and overall FA for FA based predictors or overall MD for MD based predictors | | | | | | | | | | | | | | |

| Subgroup |  |  | Number of CDS symptoms | | | | | |
| --- | --- | --- | --- | --- | --- | --- | --- | --- |
|  |  |  | beta | | 95% CI | | | p |
| Patients without dementia (n=36) | WMH | NbM overlap volume | -0.07 | -1.25 | | 1.12 | 0.91 | |
|  |  | - Proximal | -0.35 | -1.46 | | 0.75 | 0.53 | |
|  |  | - Intermediate | -2.71 | -6.42 | | 0.99 | 0.15 | |
|  |  | - Distal | 0.26 | -0.47 | | 1.00 | 0.48 | |
|  |  | Total volume (reference) | 0.07 | -1.22 | | 1.36 | 0.91 | |
| Patients with dementia (n=51) | WMH | NbM overlap volume | 0.05 | -0.13 | | 0.23 | 0.59 | |
|  |  | - Proximal | 0.09 | -0.09 | | 0.27 | 0.32 | |
|  |  | - Intermediate | 0.04 | -0.14 | | 0.22 | 0.66 | |
|  |  | - Distal | 0.01 | -0.19 | | 0.21 | 0.92 | |
|  |  | Total volume (reference) | 0.03 | -0.15 | | 0.22 | 0.72 | |
| **Supplementary Table 4 II: dementia subgroup WMH analysis.** Poisson linear regression of total number of cholinergic deficiency syndrome (CDS) symptoms predicted by the overlap between white matter hyperintensities (WMH) and the listed regions separately in patients with and without a diagnosis of dementia. NbM: total nucleus basalis of Meynert (NbM) cortical pathway. Proximal: proximal part only. Intermediate: intermediate part only. Distal: distal part only. Total volume: total brain WMH volume | | | | | | | | |

|  |  | > 1 symptom of CDS | | | | | | | | | | | |
| --- | --- | --- | --- | --- | --- | --- | --- | --- | --- | --- | --- | --- | --- |
|  |  | model 2 (adjusted) * | | | | | model 3 (additionally adjusted) * | | | | | | |
|  |  | OR | 95% CI | | p | | OR | | | 95% CI | | | P |
| FA | NbM | -0.22 | -0.44 | 0.00 | | 0.05 | | -0.20 | -0.45 | | 0.04 | 0.10 | |
|  | - Proximal | -0.24 | -0.45 | -0.03 | | 0.03 | | -0.24 | -0.47 | | -0.01 | 0.04 | |
|  | - Intermediate | -0.28 | -0.48 | -0.07 | | 0.01 | | -0.27 | -0.51 | | -0.04 | 0.02 | |
|  | - Distal | -0.14 | -0.37 | 0.09 | | 0.22 | | -0.11 | -0.36 | | 0.14 | 0.39 | |
|  | Whole brain (reference) | 0.00 | -0.24 | 0.24 | | 0.99 | | -0.02 | -0.26 | | 0.23 | 0.90 | |
| MD | NbM | 0.32 | 0.01 | 0.63 | | 0.05 | | 0.34 | 0.00 | | 0.68 | 0.05 | |
|  | - Proximal | 0.28 | 0.01 | 0.55 | | 0.04 | | 0.34 | 0.03 | | 0.65 | 0.03 | |
|  | - Intermediate | 0.30 | 0.06 | 0.55 | | 0.02 | | 0.35 | 0.08 | | 0.63 | 0.01 | |
|  | - Distal | 0.16 | -0.05 | 0.38 | | 0.14 | | 0.16 | -0.07 | | 0.39 | 0.18 | |
|  | Whole brain (reference) | 0.21 | 0.04 | 0.38 | | 0.02 | | 0.20 | 0.01 | | 0.39 | 0.04 | |
| **Supplementary Table 4 III: additionally adjusted analysis.** Poisson linear regression of total number of cholinergic deficiency syndrome (CDS) symptoms predicted by the fractional anisotropy (FA) and mean diffusivity (MD) in the listed regions. NbM: total nucleus basalis of Meynert cortical pathway, Proximal: proximal part only, Intermediate: intermediate part only, Distal: distal part only, *model 2: adjusted for atrophy and overall FA for FA based predictors or overall MD for MD based predictors | | | | | | | | | | | | | |

|  |  | Cerebral atrophy | | | | | | | | | | | |
| --- | --- | --- | --- | --- | --- | --- | --- | --- | --- | --- | --- | --- | --- |
|  |  | model 1 (unadjusted) * | | | | | model 2 (adjusted) * | | | | | | |
|  |  | OR | 95% CI | | p | | OR | | | 95% CI | | | P |
| FA | NbM | -0.50 | -0.79 | -0.21 | | 0.001 | | -0.38 | -0.68 | | -0.08 | 0.01 | |
|  | - Proximal | -0.47 | -0.76 | -0.18 | | 0.003 | | -0.34 | -0.65 | | -0.02 | 0.04 | |
|  | - Intermediate | -0.49 | -0.78 | -0.19 | | 0.002 | | -0.35 | -0.67 | | -0.04 | 0.03 | |
|  | - Distal | -0.44 | -0.74 | -0.14 | | 0.005 | | -0.30 | -0.62 | | 0.03 | 0.07 | |
|  | Whole brain (reference) | 0.45 | 0.16 | 0.75 | | 0.004 | | 0.31 | 0.01 | | 0.61 | 0.044 | |
| MD | NbM | 0.46 | 0.16 | 0.75 | | 0.003 | | 0.71 | 0.33 | | 1.08 | 0.001 | |
|  | - Proximal | 0.41 | 0.10 | 0.71 | | 0.01 | | 0.78 | 0.35 | | 1.21 | 0.001 | |
|  | - Intermediate | 0.46 | 0.16 | 0.76 | | 0.003 | | 0.56 | 0.22 | | 0.9 | 0.002 | |
|  | - Distal | 0.56 | 0.29 | 0.84 | | <0.001 | | 0.58 | 0.29 | | 0.86 | <0.001 | |
|  | Whole brain (reference) | 0.09 | -0.25 | 0.42 | | 0.61 | | -0.38 | -0.76 | | 0.00 | 0.05 | |
| **Supplementary Table 4 IV: cerebral atrophy in patients with Alzheimer’s Disease:** Linear regression of standardized cerebral atrophy predicted by the standardized fractional anisotropy (FA) and mean diffusivity (MD) in the listed regions, in patients with Alzheimer’s Disease. Cerebral atrophy was operationalized as 1 minus the total brain volume divided by the intra cranial volume. NbM: total nucleus basalis of Meynert cortical pathway, Proximal: proximal part only, Intermediate: intermediate part only, Distal: distal part only, *model 2: adjusted for atrophy and overall FA for FA based predictors or overall MD for MD based predictors | | | | | | | | | | | | | |

|  |  | MMSE score | | | | | | | | | | | |
| --- | --- | --- | --- | --- | --- | --- | --- | --- | --- | --- | --- | --- | --- |
|  |  | model 1 (unadjusted) * | | | | | model 2 (adjusted) * | | | | | | |
|  |  | OR | 95% CI | | p | | OR | | | 95% CI | | | P |
| FA | NbM | 0.02 | -0.32 | 0.35 | | 0.92 | | -0.06 | -0.42 | | 0.30 | 0.75 | |
|  | - Proximal | 0.01 | -0.33 | 0.34 | | 0.97 | | -0.09 | -0.46 | | 0.28 | 0.64 | |
|  | - Intermediate | 0.00 | -0.33 | 0.34 | | 0.99 | | -0.09 | -0.46 | | 0.28 | 0.62 | |
|  | - Distal | 0.04 | -0.29 | 0.37 | | 0.80 | | -0.05 | -0.42 | | 0.33 | 0.80 | |
|  | Whole brain (reference) | -0.17 | -0.50 | 0.15 | | 0.29 | | -0.20 | -0.56 | | 0.16 | 0.28 | |
| MD | NbM | -0.13 | -0.46 | 0.20 | | 0.44 | | -0.10 | -0.55 | | 0.34 | 0.65 | |
|  | - Proximal | -0.05 | -0.38 | 0.29 | | 0.78 | | 0.07 | -0.43 | | 0.58 | 0.78 | |
|  | - Intermediate | -0.04 | -0.37 | 0.30 | | 0.83 | | 0.02 | -0.37 | | 0.41 | 0.91 | |
|  | - Distal | -0.17 | -0.49 | 0.16 | | 0.31 | | -0.15 | -0.49 | | 0.19 | 0.39 | |
|  | Whole brain (reference) | -0.10 | -0.44 | 0.23 | | 0.53 | | -0.04 | -0.48 | | 0.41 | 0.86 | |
| **Supplementary Table 4 V: MMSE score in patients with Alzheimer’s Disease:** Linear regression of standardized MMSE score predicted by the standardized fractional anisotropy (FA) and mean diffusivity (MD) in the listed regions in patients with Alzheimer’s Disease. NbM: total nucleus basalis of Meynert cortical pathway, Proximal: proximal part only, Intermediate: intermediate part only, Distal: distal part only, *model 2: adjusted for atrophy and overall FA for FA based predictors or overall MD for MD based predictors, model 3: additionally adjusted for age, sex and medication potentially interfering with the number of neuropsychiatric symptoms. | | | | | | | | | | | | | |

|  |  | **mortality** | | | | | | | |
| --- | --- | --- | --- | --- | --- | --- | --- | --- | --- |
|  |  | **model 1 (unadjusted)** | | | | **model 2 (adjusted)*** | | | |
|  |  | **HR** | **95% CI** | | **p** | **HR** | **95% CI** | | **P** |
| FA | NbM | 0.60 | 0.35 | 1.04 | 0.07 | 0.63 | 0.33 | 1.2 | 0.16 |
|  | - Proximal | 0.57 | 0.34 | 0.94 | 0.03 | 0.57 | 0.32 | 1.02 | 0.06 |
|  | - Intermediate | 0.67 | 0.42 | 1.06 | 0.08 | 0.65 | 0.34 | 1.24 | 0.19 |
|  | - Distal | 0.61 | 0.38 | 0.97 | 0.03 | 0.56 | 0.29 | 1.07 | 0.08 |
|  | Whole brain (reference) | 1.73 | 1.01 | 2.96 | 0.05 | 1.46 | 0.78 | 2.73 | 0.23 |
| MD | NbM | 1.49 | 0.91 | 2.45 | 0.12 | 0.89 | 0.41 | 1.93 | 0.77 |
|  | - Proximal | 1.53 | 1.02 | 2.31 | 0.04 | 1.24 | 0.60 | 2.58 | 0.56 |
|  | - Intermediate | 1.34 | 0.82 | 2.20 | 0.24 | 0.84 | 0.41 | 1.69 | 0.62 |
|  | - Distal | 1.33 | 0.86 | 2.04 | 0.20 | 1.05 | 0.54 | 2.04 | 0.89 |
|  | Whole brain (reference) | 1.50 | 0.98 | 2.30 | 0.06 | 1.57 | 1.00 | 2.46 | 0.05 |
| **Supplementary Table 4 VI: mortality risk.** Cox proportional hazard model of 3-year mortality (n=86, events=13) predicted by the standardized fractional anisotropy (FA) and mean diffusivity (MD) in the listed regions. HR: standardized hazard ratio, NbM: total nucleus basalis of Meynert cortical pathway, Proximal: proximal part only, Intermediate: intermediate part only, Distal: distal part only. *adjusted for cerebral atrophy and whole brain FA for FA based predictors or whole brain MD for MD based predictors. | | | | | | | | | |
